# Deep Learning-Based Instance-Level Segmentation of Kidney and Liver Cysts in Computed Tomography Images of Patients Affected by Polycystic Kidney Disease

**DOI:** 10.34067/KID.0000000924

**Published:** 2025-08-14

**Authors:** Adriana V. Gregory, Muhammed Khalifa, Jeeho Im, Sumana Ramanathan, Doaa E. Elbarougy, Conrad Cruz, Hana Yang, Aleksandar Denic, Andrew D. Rule, Fouad T. Chebib, Neera K. Dahl, Marie C. Hogan, Peter C. Harris, Vicente E. Torres, Bradley J. Erickson, Theodora A. Potretzke, Timothy L. Kline

**Affiliations:** 1 Department of Radiology, Mayo Clinic, Rochester, Minnesota; 2 Division of Nephrology and Hypertension, Mayo Clinic, Rochester, Minnesota; 3 Division of Nephrology and Hypertension, Mayo Clinic, Jacksonville, Florida

**Keywords:** ADPKD, cystic kidney, kidney volume, liver cysts, polycystic kidney disease, artificial intelligence, biomarkers

## Abstract

**Key Points:**

- Automated cyst segmentation in autosomal dominant polycystic kidney disease could transform care by enabling precise monitoring of cyst progression with minimal manual effort.
- This technology can facilitate more consistent assessments of cyst burden across patients.
- Translating these segmentation models from magnetic resonance imaging to computed tomography is crucial for providing comprehensive, modality-independent evaluations.

**Background:**

Total kidney and liver volumes are key image-based biomarkers to predict the severity of kidney and liver phenotype in autosomal dominant polycystic kidney disease (ADPKD). However, magnetic resonance (MR) imaging-based advanced biomarkers like total cyst number and cyst parenchyma surface area have been shown to more accurately assess cyst burden and improve the prediction of disease progression. The main aim of this study was to extend the calculation of advanced biomarkers to other imaging modalities; thus, we propose a fully automated model to segment kidney and liver cysts in computed tomography (CT) images.

**Methods:**

Abdominal CTs of patients with ADPKD were gathered retrospectively between 2001 and 2018. A three-dimensional deep learning method using the nnU-Net architecture was trained to learn cyst edges-cores and the noncystic kidney/liver parenchyma. Separate segmentation models were trained for kidney cysts in contrast-enhanced CTs and liver cysts in noncontrast CTs using an active learning approach. Two experienced research fellows manually generated the reference standard segmentation, which were reviewed by an expert radiologist for accuracy.

**Results:**

Two-hundred CT scans from 148 patients (mean age, 51.2±14.1 years; 48% male) were used for model training (80%) and testing (20%). In the test set, both models showed good agreement with the reference standard segmentations, similar to the agreement between two independent human readers (model versus reader: TCN_kidney/liver_ *r*=0.96/0.97 and CPSA_kidney_ *r*=0.98, inter-reader: TCN_kidney/liver_ *r*=0.96/0.98 and CPSA_kidney_ *r*=0.99).

**Conclusions:**

Our study demonstrates that automated models can segment kidney and liver cysts accurately in CT scans of patients with ADPKD.

## Visual Abstract

**Figure:**
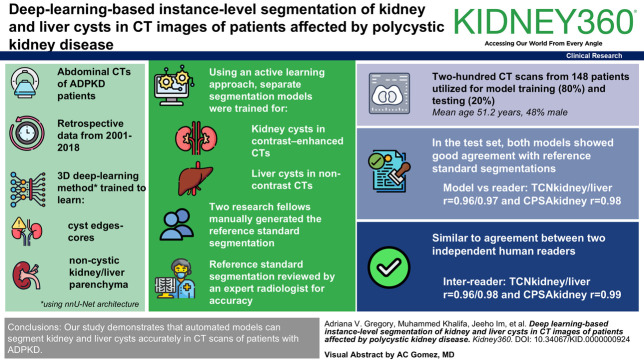

## Introduction

Autosomal dominant polycystic kidney disease (ADPKD) is the most common monogenic kidney disease, characterized by cyst formation and kidney enlargement and may ultimately lead to a progressive decline in kidney function.^1^ In addition, ADPKD is a systemic disorder that causes extrarenal cystic proliferation and manifestations. Over 90% of patients with ADPKD over 35 years of age develop liver cysts, which can lead to polycystic liver disease (PLD).^2^ PLD can cause various complications^3^ and the treatment of choice is greatly contingent upon the cysts' location and size, requiring radiological identification of the targeted cyst.^4^

Therefore, it is crucial to have biomarkers able to detect early signs of disease progression, provide relevant information for surgical planning, and monitor response to therapy and intervention. Total kidney volume (TKV) has been used as the most established prognostic imaging biomarker to predict ADPKD progression.^5^ Total liver volume (TLV) is noted as a biomarker for PLD.^6^ However, these biomarkers have many limitations. For instance, patients with similar TKVs may exhibit different clinical presentations and phenotypes; on the other hand, TLV does not specifically distinguish between cysts that are suitable for treatments like sclerotherapy. Hence, developing more advanced biomarkers is still required to provide a better assessment of the cyst burden and improve the current clinical management.

Kidney and liver segmentation using deep learning (DL) has been explored in recent years in magnetic resonance (MR) and computed tomography (CT) images from patients affected by ADPKD.^7–17^ Fully automated convolutional neural networks using the U-Net architecture have been the most successful models. The automated segmentation of polycystic kidneys has achieved a Dice score of up to 0.97^11^ and polycystic livers a Dice score of 0.95,^17^ where a Dice score of 1.00 indicates perfect agreement between reference standard and the predicted segmentation. Later, recognizing the importance of assessing separately cyst volume to better understand disease progression,^18,19^ studies investigated the use of DL in cyst segmentation. Kline *et al.* proposed an automated approach for semantic segmentation of kidney cysts in MR images of patients with ADPKD achieving a Dice score of 0.86.^20^ Subsequently, Schmidt *et al.* studied the importance of having a diverse dataset in building DL-models (*i.e*., single institution data versus multi-institution data). For kidney cyst segmentation in MR images, the Dice score by the model trained with all institutional data ranged from 0.83 to 0.89, concluding that segmentation accuracy increases with models trained on larger sample sizes, especially in more complex segmentation tasks.^21^ In MR liver cyst segmentation, Chookhachizadeh *et al.* trained a DL-model that achieved a Dice score ranging from 0.8 to 0.82 denoting that the model reduced the annotation time by 91%.^22^ Previous studies further identified total cyst number (TCN) as a potential image biomarker that could enhance the prediction of disease progression.^23,24^ The image processing domain of instance-based segmentation can help distinguish individual instances of the same class, offering a higher level of detail than semantic segmentation alone.^25^ Gregory *et al.* developed an automated approach for three dimensional (3D)-instance segmentation of kidney cysts in T2-MR images for patients with ADPKD, achieving an average Dice score of 0.85.^26^ In a subsequent study using the Consortium of Radiologic Imaging Study of Polycystic Kidney Disease (PKD) dataset, it was determined that TCN and cyst-parenchyma surface area (CPSA) have superior performance compared with TKV in the prediction of eGFR decline, kidney failure, and CKD stages 3A, 3B, and 4.^5^ However, to date, there is a lack of studies focused on cyst segmentation in CT images of patients with ADPKD.

Although MR imaging is generally the preferred imaging modality for comprehensive ADPKD management because of its accuracy, ability to characterize cysts, and safety, CT imaging is often ordered for specific assessments. It is particularly valuable for detecting kidney stones, calcifications, infections, cyst hemorrhage, and suspicious masses.^27^ In addition, renal cysts and polycystic kidneys are often discovered in CT images incidentally during the evaluation of abdominal pain. Therefore, automated methods to segment individual kidney and liver cysts in CT images that produce accurate cyst quantification parameters are still needed. We propose the use of DL to produce 3D-instance cyst segmentation models to segment kidney and liver cysts in CT images to enable the calculation of advanced biomarkers for CT imaging.

## Methods

### Dataset Overview

This is a retrospective imaging study reviewed and approved by our Institutional Review Board (IRB). IRB approval included a waiver of patient informed consent. Abdominal CT scans from patients affected by ADPKD were searched in our Radiology Database between 2001 and 2018. Only axial CT scans were included in the study; the exclusion criteria were scans presenting incomplete liver or kidney coverage, nephrectomies, kidney allografts, and imaging artifacts.

### Data Curation

The low dimensionality representations of the imaging dataset generated using FiftyOne^28^ helped visualize and exclude the examinations that met the exclusion criteria and to sort contrast-enhanced versus noncontrast CT images. While non–contrast-enhanced CT scans provide good visualization of liver cysts, cysts in the kidneys (particularly small cysts) are more difficult to identify unless a contrast media is applied. Thus, 100 contrast-enhanced (for kidney) and 100 noncontrast CT scans (for liver) were selected using systematic sampling on the datasets sorted by TKV and TLV, respectively, to ensure representation of cases with varying degrees of cystic disease. Twenty cases, ranging from relatively limited cystic involvement to more advanced polycystic disease, were selected from each kidney and liver dataset as a test set on the basis of TKV/TLV and visual inspection of cyst burden. The remaining 80 cases in each set were used for training the segmentation models. Kidney and liver volumes were calculated using the model by Kline *et al*.^29^

### Active Learning Training Strategy and Data Labeling

**Figure 1:**
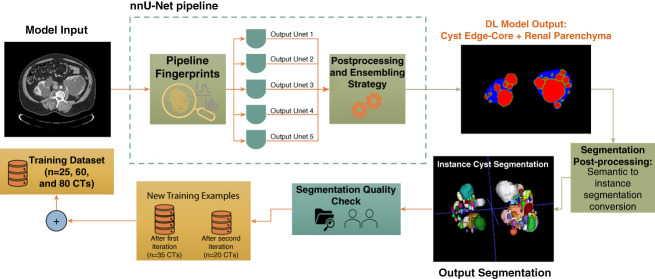
**DL-model active learning workflow.** The diagram applies to both the kidney-cyst and the liver-cyst segmentation models. The nnU-Net network was first trained using 25 CT scans; the input to the network is one complete CT volume. Five three dimensional Unets were trained using different splits of the training set (*i.e*., five-fold cross-validation). The predictions were ensembled using the nnU-Net pipeline. The output of the model is a semantic segmentation mask with the same shape as the input image, containing three labels: cyst-core (red), cyst-edge (green), and kidney/liver parenchyma (blue). A postprocessing step is applied to convert the semantic segmentation to an instance-cyst segmentation. The output segmentations were reviewed for quality by two expert research fellows. The second model retraining included 35 additional CT scans for a total of 60 CT training examples. The last model training iteration included 20 additional CT scans for a total of 80 CT training examples. CT, computed tomography; DL, deep learning.

Two models were trained to predict kidney cysts in contrast-enhanced CTs and liver cysts in noncontrast CTs. Each model was trained using an active learning workflow. The workflow included three training stages with training sets of 25, 60, and 80 CTs and two prediction quality assessment points between the training stages (Figure 1). Details of the training stages are presented in Supplemental Materials 1.1.

The segmentations were labeled by two trained image annotators and checked for quality by an expert radiologist using an in-house developed polycystic kidney disease-graphical user interface software with custom designed functions to easily label, split, and merge cysts. In total, 120 cases (75%) of the training set were labeled by the most senior annotator and 40 cases (25%) by the second annotator. In the test sets, the two independent annotators reviewed each case separately. The segmentations from the least experienced annotator were further checked by an expert radiologist (*i.e*., reference standard segmentation) and are referred to as reader 1 or expert reader in the results. The second reader is referred to as reader 2.

### DL Model (nnU-Net)

The DL architecture used for this study was the nnU-Net version 2.^30^ This is a semantic segmentation model that can be rapidly adapted to any given dataset. The workflow automatically analyzes the training dataset and configures a U-Net-based segmentation pipeline appropriate for the specific dataset used. A 3D-Unet which uses high resolution images was configured. We used an Nvidia graphical processing unit (model: A100-SXM4) and Python 3.9. The nnU-Net models were trained with five-folds. The prediction was obtained after a postprocessing step (configured by the nnU-Net pipeline) which ensembles all the folds. Each model was trained for 1000 epochs with learning-rate decay. The data preprocessing (Supplemental Figure 1) before training and output postprocessing (Supplemental Figure 2) methods are described in Supplemental Materials 1.2 and 1.3.

### Statistical Analysis

The accuracy of the DL-models to measure total cyst volume (TCV), TCN, and CPSA was assessed. For liver cysts, only TCV and TCN were calculated because the liver cysts are endophytic. Kidney cysts can be either exophytic or endophytic. Exophytic cysts grow outwards, likely having a lower effect on the kidney parenchyma compared with endophytic cysts. CPSA can measure this effect because it is defined as the sum of all cyst surfaces minus the outer surface of exophytic cysts. The predicted 8-year slope of eGFR on the basis of TCN and CPSA was calculated for the kidney results using the formulas^5^:


8 year Slope of eGFR=−0.792−0.007×TCN

8 year Slope of eGFR=−1.075−0.002×CPSA.

**Figure 2:**
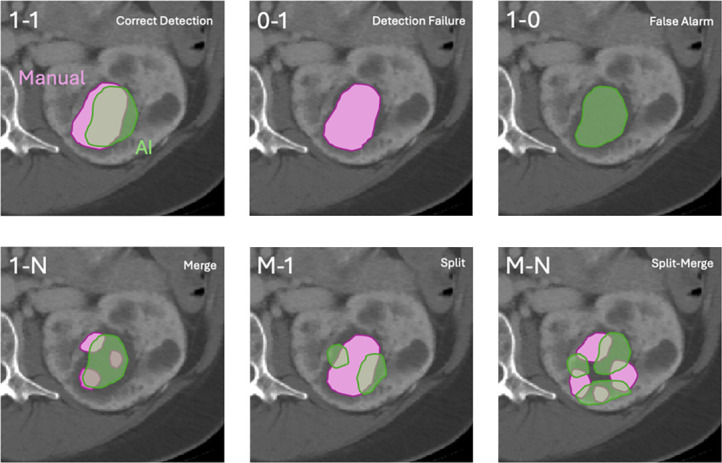
**Figure illustrates different types of correspondences, in this example, between manually segmented cysts (depicted in purple) and automatically segmented cysts (depicted in green) when performing instance cyst segmentation comparisons.** The various categories include 1–1: a correct detection where there is a one-to-one correspondence between a manual cyst and an automatic cyst with significant overlap; 0–1: a false positive, where the automatic segmentation detects a cyst that is not present in the manual segmentation; 1–0: a false negative, where a cyst is present in the manual segmentation but not detected in the automatic segmentation; 1–N: a split scenario where a single cyst from the manual segmentation is incorrectly split into multiple smaller cysts by the automatic segmentation; M–1: a merge scenario where multiple distinct cysts from the manual segmentation are incorrectly merged into a single cyst by the automatic segmentation; M–N: a complex split-merge scenario where multiple cysts in the manual segmentation correspond to multiple cysts in the automatic segmentation with overlapping and mismatches in both directions.

Linear regression and Bland–Altman plots were used to measure correlation, bias, and limits of agreement. Similarity metrics including Dice score and Jaccard index were used to evaluate segmentation overlap agreement between predicted and manual segmentations. Furthermore, we used the Nascimento nomenclature^31^ to compute cyst-level: correct detections, false positives, false negatives, splits, merges, and split-merges (Figure 2).^32^ The methods for calculating the Nascimento metrics are described in Supplemental Materials 1.4. In addition, inter-reader comparisons were performed to assess the agreement between the two readers.

## Results

### Study Sample

**Figure 3:**
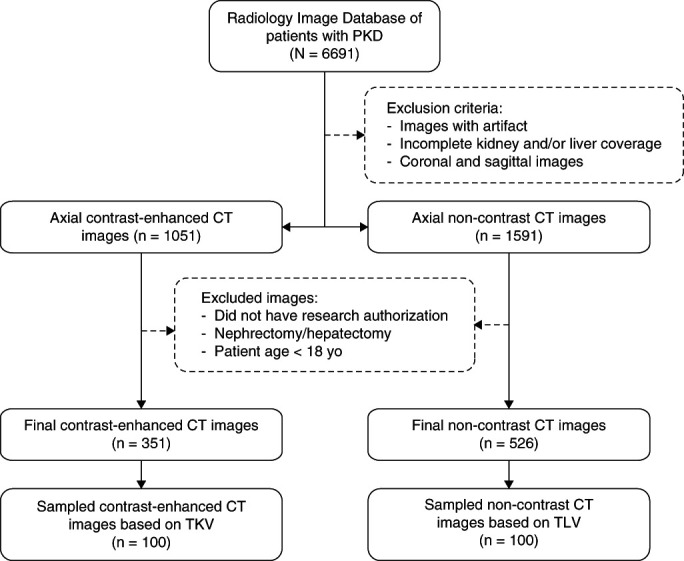
**Selection of the study population to develop the kidney and liver cyst instance segmentation DL-models.** PKD, polycystic kidney disease; TKV, total kidney volume; TLV, total liver volume.

A total of 6691 CT scans from 1842 patients with ADPKD were found in our radiology image database. After the exclusion criteria, a total of 351 contrast-enhanced CT scans with complete kidney coverage and 526 noncontrast CT scans with complete liver coverage were identified. Representative subsets of 100 contrast-enhanced and 100 noncontrast CT scans were used to build DL-models that segment cysts individually in kidneys and liver, respectively (Figure 3). The characteristics of the patients and CT scans included in the study are shown in Table 1. The number of training/testing cyst examples were 15,820/3965 and 11,982/3866 in the kidney and liver cohorts, respectively.

**Table 1:** Study population and computed tomography image characteristics

Patient and Imaging Characteristics	Kidney Images Train Set (*n*=80)	Kidney Images Test Set (*n*=20)	Liver Images Train Set (*n*=80)	Liver Images Test Set (*n*=20)
**Demographics**				
Age, mean (SD), yr	51.4 (15.2)	50.2 (13.2)	51.7 (13.1)	51.6 (11.5)
Men, *n* (%)	31 (39)	9 (45)	48 (60)	8 (40)
Race, *n* (%)				
*American Indian/Alaskan Native*	—	2 (10)	—	—
*Asian*	2 (2.5)	2 (10)	—	1 (5)
*Black or African American*	—	—	—	2 (10)
*Other/unknown*	7 (8.75)	2 (10)	3 (3.75)	—
*White*	71 (88.75)	14 (70)	77 (96.25)	17 (85)
CKD				
*Stage 1*	19 (24)	8 (40)	6 (8)	2 (5)
*Stage 2*	38 (48)	7 (35)	14 (18)	2 (10)
*Stage 3a*	15 (19)	1 (5)	13 (16)	1 (5)
*Stage 3b*	3 (4)	2 (10)	12 (15)	5 (25)
*Stage 4*	1 (1)	—	17 (21)	9 (45)
*Stage 5*	2 (3)	1 (5)	13 (16)	
*Not available*	2 (3)	1 (5)	5 (6)	1 (5)
Genotype and effect				
*PKD1 truncating*	14 (18)	1 (5)	21 (26)	6 (30)
*PKD1 nontruncating*	5 (6)	5 (25)	13 (16)	4 (20)
*PKD2 truncating*	8 (10)	—	8 (10)	2 (10)
*PKD2 nontruncating*	2 (3)	—	5 (6)	—
*Other^a^*	5 (6)	—	3 (4)	—
*Not available*	46 (58)	14 (70)	30 (38)	8 (40)
Mayo imaging classification				
*1A*	14 (18)	6 (30)	—	—
*1B*	22 (28)	6 (30)	—	—
*1C*	25 (31)	3 (15)	—	—
*1D*	5 (6)	1 (5)	—	—
*1E*	2 (3)	1 (5)	—	—
*2A*	9(11)	2 (10)	—	—
*2B*	1(1)	—	—	—
*Not available*	2 (3)	1 (5)	—	—
**CT organ automated segmentation, cc, median (range)**				
Kidney cohort TKV	888 (289–2749)	875 (372–3315)	—	—
Liver cohort TLV	—	—	2098 (1088–7748)	1805 (977–3450)
**CT scanner manufacturer, *n* (%)**				
Fujifilm	1 (1.25)	—	—	—
GE Medical Systems	7 (12.5)	5 (25)	15 (18.75)	5 (25)
Imatron	1 (1.25)	—	—	—
Siemens	65 (81.25)	14 (70)	55 (68.75)	11 (55)
Toshiba	6 (7.5)	1 (5)	10 (12.5)	4 (20)
**CT image resolution, mm, median (range), mm**				
Pixel spacing	0.74 (0.59–0.98)	0.74 (0.62–0.98)	0.78 (0.62–0.97)	0.74 (0.64–0.93)
Slice thickness	2.5 (0.8–5)	3 (1–5)	5 (1.5–5)	5 (2.5–5)

Data are presented as mean (SD), *n* (%), or median (range).

Race is self-reported.

CKD stages calculated using the formula CKD Epidemiology Collaboration 2021 and serum creatinine within 60 days from imaging date. CT, computed tomography; PKD, polycystic kidney disease; TKV, total kidney volume; TLV, total liver volume.

a Other genotypes include *ALG8*, *ALG9*, *PRKCSH*, *IFT140*.

### Semantic Kidney Cyst Segmentation Results

In the kidney test set (*n*=20), the segmentations from reader 1 and reader 2 had a higher overlap agreement (average Dice=0.89) compared with each reader with the DL-model (average Dice of 0.85 and 0.83, respectively). The inter-reader agreement in TCV_kidney_ (bias=−10.2%; *r*=1.0) was slightly better compared with the agreement between reader 1 and the DL-model (bias=−14.2%; *r*=1.0), but lower compared with the agreement between reader 2 and the DL-model (bias=−3.9%; *r*=0.99), and between the average of the readers and the DL-model (bias=−8.7; *r*=0.99).

### Individual Kidney Cyst Segmentation Results

**Figure 4:**
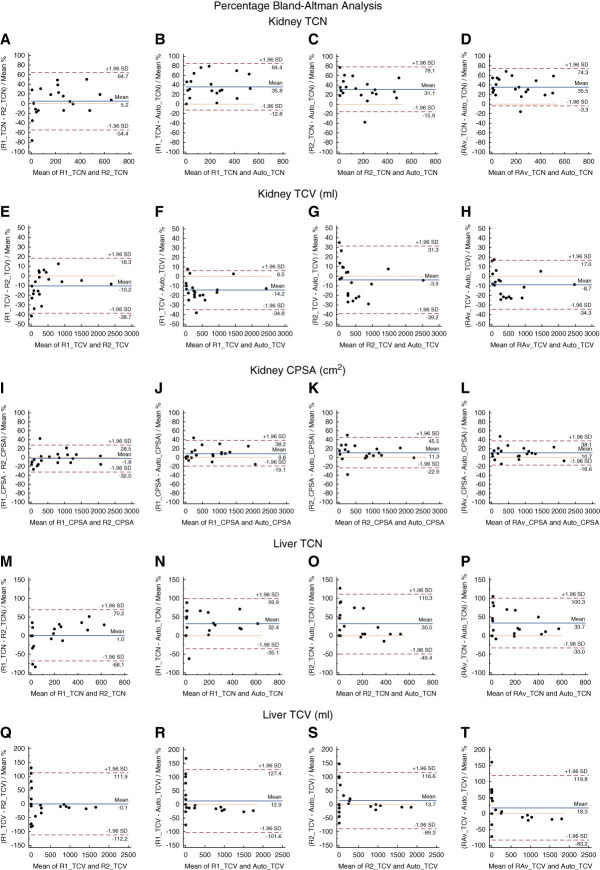
**Bland–Altman plots assessing the inter-reader agreement (first column), the expert reader and the DL-model agreement (second column), the second reader and the DL-model agreement (third column), and the readers' average with the DL-model agreement (fourth column).** These comparisons were performed for the advanced imaging biomarkers: (A–D) kidney TCN, (E–H) kidney TCV, (I–L) kidney CPSA, (M–P) liver TCN, and (Q–T) liver TCV. Visual assessment shows similar bias and limits of agreement for each biomarker comparisons. CPSA, cyst-parenchyma surface area; TCN, total cyst number; TCV, total cyst volume.

**Figure 5:**
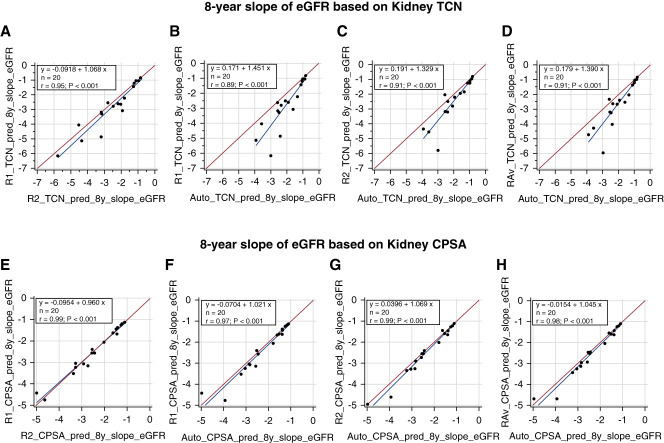
**Linear regression plots showing a high linear relationship between the reader-model predicted 8-year slope of eGFR based on TCN, and CPSA.** Plots (A and E) show the inter-reader agreement, plots (B and F) show the agreement between the expert reader and the DL-model, plots (C and G) show the agreement between the second reader and the DL-model, and plots (D and H) show the agreement between the average of the readers and the DL-model.

The agreement in TCN_kidney_ between the readers (bias=5.2%; *r*=0.96) was better compared with the agreement of reader 2 and reader 1 with the DL-model (bias=31.1% and 35.8%; *r*=0.95 and 0.94, respectively) and compared with the average of the readers with the DL-model (bias=35.5%; *r*=0.96). The inter-reader agreement in CPSA_kidney_ (bias=−1.8%; *r*=0.99) was slightly better compared with the agreement between reader 1 and the DL-model (bias=9.6%; *r*=0.97), the agreement between reader 2 and the DL-model (bias=11.3%; *r*=0.99), and the agreement between the average of the readers and the DL-model (bias=10.7; *r*=0.98). A summary of these results is presented in Table 2. The Bland–Altman plots showing the kidney cyst percentage bias and limits of agreement are shown in Figure 4. Negative bias indicates overestimation by the DL-model. The linear regression and absolute-bias Bland–Altman plots are shown in Supplemental Figures 3–5. In Figure 5, the slope of eGFR calculated by CPSA showed stronger agreements compared with the slope of eGFR calculated by TCN, suggesting that CPSA might be a more stable biomarker.

**Table 2:** Similarity analysis: inter-reader and reader versus deep learning model

Similarity Metrics	Reader 1 versus Reader 2	Reader 1 versus DL-Model	Reader 2 versus DL-Model	Reader's Average versus DL-Model
**Kidney**				
Dice score	0.89±0.07	0.83±0.06	0.85±0.07	—
Jaccard index	0.82±0.11	0.71±0.09	0.74±0.11	—
Precision	0.94±0.05	0.89±0.07	0.87±0.09	—
Recall	0.86±0.11	0.78±0.07	0.84±0.1	—
TCV_kidney_ (bias±SD), %	−10.2±14.5	−14.2±10.5	−3.9±17.9	−8.7±13.1
TCV_kidney_ (*r*)	1.00^a^	1.00^a^	0.99^a^	0.99^a^
TCN_kidney_ (bias±SD), %	5.2±30.4	35.8±24.8	31.1±24	35.5±19.8
TCN_kidney_ (*r*)	0.96^a^	0.94^a^	0.95^a^	0.96^a^
CPSA_kidney_ (bias±SD), %	−1.8±15.5	9.6±14.6	11.3±17.4	10.7±14
CPSA_kidney_ (*r*)	0.99^a^	0.97^a^	0.99^a^	0.98^a^
**Liver**				
Dice score	0.70±0.2	0.72±0.2	0.68±0.25	—
Jaccard index	0.6±0.2	0.6±0.2	0.56±0.27	—
Precision	0.73±0.2	0.78±0.15	0.72±0.15	—
Recall	0.75±0.26	0.71±0.24	0.68±0.3	—
TCV_liver_ (bias±SD), %	−0.1±57.1	12.9±58.4	13.7±52.5	18.3±51.8
TCV_liver_ (*r*)	1.00^a^	1.00^a^	1.00^a^	1.00^a^
TCN_liver_ (bias±SD), %	1.0±35.3	32.4±34.4	30.5±40.7	33.7±34
TCN_liver_ (*r*)	0.98^a^	0.96^a^	0.96^a^	0.97^a^

Data are presented as mean±SD. CPSA, cyst-parenchyma surface area; DL, deep learning; TCN, total cyst number; TCV, total cyst volume.

a *P* < 0.001.

**Figure 6:**
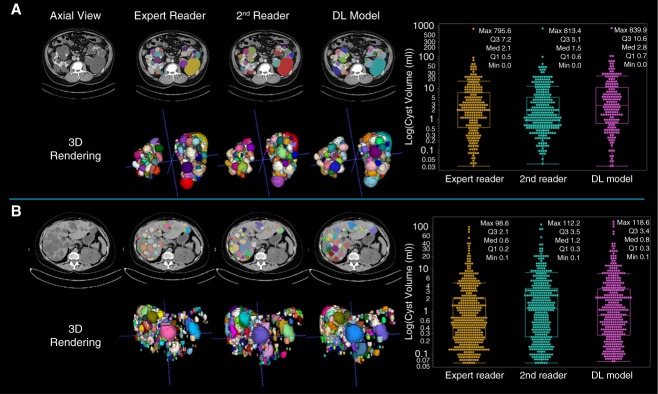
**Example images.** (A) Axial contrast-enhanced CT image of a 65-year-old male patient presenting with multiple bilateral kidney cysts. The manual segmentation by the expert reader resulted in a TCV_kidney_ of 2280 ml and a TCN_kidney_ of 246 cysts. The segmentations performed by the second reader resulted in a TCV_kidney_ of 2477 ml and a TCN_kidney_ of 283 cysts. The segmentations predicted by the DL-model resulted in a TCV_kidney_ of 2581 ml and a TCN_kidney_ of 185 cysts. (B) Axial noncontrast CT image of a 64-year-old female patient presenting with multiple liver cysts. The manual segmentation by the expert reader resulted in a TCV_liver_ of 1292 ml and a TCN_liver_ of 517 cysts. The segmentations performed by the second reader resulted in a TCV_liver_ of 1528 ml and a TCN_liver_ of 447 cysts. The segmentations predicted by the DL-model resulted in a TCV_liver_ of 1698 ml and a TCN_liver_ of 431 cysts. The color of the cysts are assigned randomly and are not expected to be the same between the different segmentations. The box plots depict the individual cyst volumes calculated from the segmentation performed by the expert reader, reader 2, and the DL model. For each box plot, the minimum, maximum, interquartile range, and median are displayed to provide a more detailed summary of the distribution. 3D, three dimensional.

In the Nascimento nomenclature analysis of kidney cysts, when comparing the DL-model with reader 1, there were 1836 correct cyst detections, with a mean of 91.8±76.7 per case. The number of false positives was 372 (18.6±22.7 per case), while false negatives totaled 1876 (93.8±94.5 per case). Splits and merges totaled 111 and 338, respectively, and the presence of split-merges was moderate, with 195 occurrences (9.8±12.7 per case). When comparing the DL-model and reader 2, the correct detections were slightly lower at 1814 (90.7±78.5), with false positives totaling 367 (18.4±20.7) and false negatives reduced to 1254 (62.7±62.5). The incidence of splits increased to 148, while the number of merges was 304. Split-merges were similar to the comparison with reader 1, with a total of 190 (9.5±11.8 per case). The inter-reader comparison between reader 1 and reader 2 yielded 3639 correct detections (181.9±194.8), significantly higher than the DL comparisons. False positives and false negatives were notably lower, at 320 (16.0±16.8) and 939 (46.9±61.9), respectively. Both splits and merges were minimal, with totals of 38 and 129. Split-merges were less frequent, with only 52 occurrences (2.6±4.8). Summaries of these metrics are presented in Table 3. An example case is presented in Figure 6 showing the readers' manually segmented kidney cysts and the DL prediction.

**Table 3:** Nascimento nomenclature analysis: inter-reader and reader versus deep learning model

Comparison	Correct Detections	False Positives	False Negatives	Splits	Merges	Split-Merges
**Kidney**						
Inter-reader	3639 (181.95±194.79) (4–691)	320 (16.00±16.76) (0–70)	939 (46.95±61.95) (0–237)	38 (1.90±3.06) (0–12)	129 (6.45±8.20) (0–30)	52 (2.60±4.78) (0–19)
Reader 1 versus DL-model	1836 (91.80±76.66) (4–242)	372 (18.60±22.68) (0–83)	1876 (93.80±94.47) (0–340)	111 (5.55±6.90) (0–22)	338 (16.90±22.23) (0–91)	195 (9.75±12.72) (0–42)
Reader 2 versus DL-model	1814 (90.70±78.53) (4–258)	367 (18.35±20.67) (0–73)	1254 (62.70±62.50) (2–259)	148 (7.40±7.78) (0–22)	304 (15.20±21.51) (0–86)	190 (9.50±11.81) (0–37)
**Liver**						
Inter-reader	1707 (85.35±93.83) (0–291)	467 (23.35±21.91) (0–68)	749 (37.45±55.32) (0–203)	61 (3.05±4.06) (0–11)	374 (18.70±24.31) (0–80)	269 (13.45±20.80) (0–77)
Reader 1 versus DL-model	1382 (69.10±76.93) (0–233)	276 (13.80±16.89) (0–49)	1028 (51.40±65.76) (0–253)	121 (6.05±9.15) (0–33)	363 (18.15±24.50) (0–86)	307 (15.35±26.54) (0–99)
Reader 2 versus DL-model	1359 (67.95±73.26) (0–202)	297 (14.85±20.10) (0–55)	777 (38.85±39.92) (0–139)	213 (10.65±14.73) (0–51)	109 (5.45±6.65) (−1 to 20)	291 (14.55±27.47) (0–104)

Data are presented as total values, mean±SD, and range.

Correct detections; cysts that had a one-to-one match between the deep learning and manual segmentations on the basis of a Dice coefficient threshold of 0.1.

False positives: cysts detected by the deep learning segmentation but not present in the manual segmentation.

False negatives: cysts present in the manual segmentation but not detected by the deep learning-model.

Splits: counted when a single manual cyst was segmented as multiple cysts in the deep learning segmentation.

Merges: counted when multiple manual cysts were merged into a single cyst by the deep learning segmentation.

Split-merges: were identified when both splits and merges occurred between corresponding cysts in the deep learning and manual segmentations, with overlapping and mismatches in both directions. Corrections were made to avoid double-counting these instances. DL, deep learning.

### Semantic Liver Cyst Segmentation Results

In the liver test set (*n*=20), the segmentations from reader 1 and reader 2 had a higher overlap agreement (average Dice=0.70) compared with the agreement between reader 2 and the DL-model (average Dice=0.68) but was lower compared with the agreement between reader 1 and the DL-model (average Dice of 0.72). The TCV_liver_ agreement between the two independent readers (bias=−0.1%; *r*=1.0) had the highest agreement followed by the agreement between reader 1 and the DL-model (bias=12.9%; *r*=1.0), the agreement between reader 2 and the DL-model (bias=13.7%; *r*=1.0), and the agreement between the average of the readers and the DL-model (bias=18.3%; *r*=1.0).

### Individual Liver Cyst Segmentation Results

The inter-reader agreement in TCN_liver_ (bias=1.0%; *r*=0.98) was better compared with the agreement of reader 1 and reader 2 with the DL-model (bias=32.4% and 30.5%; *r*=0.96 and 0.96, respectively) and compared with the average of the readers with the DL-model (bias=33.7%; *r*=0.97). A summary of these results is presented in Table 2. The Bland–Altman plots showing the liver cyst percentage bias and limits of agreement are depicted in Figure 4. The linear regression and absolute-bias Bland–Altman plots are shown in Supplemental Figures 6 and 7.

In the Nascimento nomenclature analysis of liver cysts, when comparing the DL-model with reader 1 resulted in 1382 correct detections (69.1±76.9), with 276 false positives (13.8±16.9) and 1028 false negatives (51.4±65.8). The occurrence of splits and merges was higher than in the kidney, with totals of 121 and 363, respectively. Split-merges were frequent, with 307 occurrences (15.4±26.5). In the comparison between DL-model and reader 2, correct detections were similar at 1359 (67.9±73.3), while false positives and false negatives were 297 (14.9±20.1) and 777 (38.9±39.9), respectively. The number of splits increased to 213, but the number of merges reduced to 109. Split-merges remained high, totaling 291 (14.6±27.5). Finally, the inter-reader comparison showed the highest correct detection count of 1707 (85.4±93.8), with 467 false positives (23.4±21.9) and 749 false negatives (37.5±55.3). Splits and merges were less frequent than in the DL comparisons, with totals of 61 and 374, respectively. The total number of split-merges was 269 (13.5±20.8). Summaries of these metrics are presented in Table 3. An example case is presented in Figure 6 showing the readers' manually segmented liver-cysts and the DL prediction.

Given the high number of false negatives when comparing predicted cysts with corresponding manually segmented cysts but noting the high correlation in TCV, we hypothesized that most of the false negative cysts had very small individual volumes. We performed a threshold analysis and filtered cysts smaller than 1 ml. This resulted in a reduction in the false negative rate from 46% to 4%, supporting the hypothesis that most false negatives were attributable to small-volume cysts.

### Baseline Patient Characteristics and Advanced Biomarker Analysis

Model performance was evaluated across stratified patient subgroups defined by CKD stage, Mayo Imaging Classification (MIC), and TKV, to assess robustness and generalizability under varying disease severity and morphologic phenotypes. Dice score distributions stratified by CKD stage, MIC, and TKV demonstrated good to excellent agreement overall (>0.7). This indicates a robust model performance across disease severity. Greater variability in Dice scores was observed in the lower ranges of CKD stage, MIC, and TKV, which may in part reflect the larger proportion of cases with mild disease (Supplemental Figure 8).

We further evaluated the relationship between the advanced imaging biomarkers and patient subgroups defined by CKD stage and MIC, to explore potential correlations between imaging-derived metrics and disease severity. A positive association was observed between TCN and CPSA values with MIC and CKD stage, such that lower TCN and CPSA were associated with lower MIC and earlier CKD stages, while higher values corresponded to more advanced disease (Supplemental Figure 9).

## Discussion

In this study, we trained a DL approach for 3D instance segmentation of kidney and liver cysts in contrast-enhanced and noncontrast CT scans, respectively. Our results demonstrate that well stratified datasets of 100 images are large enough to train models that can perform at the level of interobserver variability. Gregory *et al.*^26^ developed a DL approach to automatically segment individual kidney cysts in T2 MR images and showed that recently identified imaging biomarkers (*i.e*., TCN and CPSA) could be more effective predictors of kidney function decline compared with TKV.^5,26,33^ Here, we propose automated instance-level cyst segmentation models able to provide advanced imaging biomarkers not only for the kidney but also for cystic livers using CT imaging. Our study is the first to develop automated approaches for individual segmentation of kidney and liver cysts in CT scans of patients affected by ADPKD. This facilitates the opportunity for additional characterization of kidney cysts in multimodal imaging studies and the assessment of cyst burden in the liver. In addition, the individual cyst volumes and cyst locations can be easily obtained and be useful for surgical planning and treatment evaluation, as in the case of foam sclerotherapy. The advanced imaging biomarkers obtained from CT scans can provide complementary information to existing MR imaging models, as in the case of retrospective longitudinal studies where various imaging modalities may have been used. Therefore, there is a pressing need to develop automated models for different imaging modalities that can facilitate the calculation of advanced cyst imaging biomarkers to provide a better assessment of the cyst burden in both kidney and liver, as well as to enable more detailed characterization of the progression of the disease.

Several approaches were adopted to improve the proposed model's performance. First, a diverse range of ADPKD phenotypes and images with varying resolutions were included in this study. In addition, our cohort encompassed liver and kidney cases with different disease severities and cyst counts, ranging from as few as ten cysts to over 1000 cysts. The final models demonstrated improved detection of cysts compared with the initial models as demonstrated by the reduction in quality review time between training stages (Supplemental Materials 2.1). A significant association was observed between the readers and between each reader with the DL-models (*P* < 0.001). Although, the results from the DL-models were comparable with the interobserver agreement, the results from the kidney model were slightly better compared with the results from the liver model (Table 2). About 50% of the liver test set was composed of cases with few and small cyst, as observed by the TCN_liver_ and TCV_liver_ in Supplemental Figures 6 and 7. Our DL-models showed low sensitivity in detecting very small cysts (<1 ml), but the disagreement was also presented in the interobserver results (Figure 4, Q–T). Smaller volumes consisting of a few voxels have less flexibility in the overlap because small misalignments or differences in identifying the cyst edges can have more severe penalties compared with larger volumes. We observed that while the percentage bias for these cases was high (Figure 4, Q–T), the absolute bias was small (Supplemental Figure 7), indicating that while the proportional difference of the biomarkers was large compared with the reference standard biomarkers, the actual difference was small. Moreover, the results from the DL-models were slightly more similar to reader 2. This might reflect the fact that the training set was mostly labeled by reader 2 (75% of the training data). However, a significant association was observed between each reader with the DL-models. Segmentation performance in the testing set was robust across disease severity metrics (*i.e*., CKD stages, MIC, and TKV), with consistently high Dice scores. The slightly greater variability observed in early disease stage cases likely reflects the presence of fewer and smaller cysts, where minor differences in segmentation can lead to lower Dice values. In addition, the observed positive relationship between TCN and CPSA with disease severity metrics (*i.e*., MIC and CKD stages) indicates that the model's advanced imaging biomarker outputs align well with known patterns of polycystic kidney disease progression.

Although no other CT studies exist to compare the cyst segmentation results from our kidney and liver models, the Dice scores achieved by our two models are on par with previously published studies done in MR imaging. In kidney cysts, semantic and instance cyst segmentation studies showed results ranging from 0.83 to 0.89,^20,21,26^ whereas in our study the Dice results ranged between 0.83 and 0.85. In liver cysts, only one study performed MR-based semantic liver cyst segmentation where the Dice score ranged between 0.8 and 0.82.^22^ In this study, the Dice score was lower, ranging between 0.68 and 0.72, and this is mostly attributable to the mild cases as discussed previously. Our model provides further delineation between adjacent liver cysts to separate and label cysts individually, providing more information than the semantic cyst segmentation. More importantly, the output of the proposed models is not intended to be used in isolation; it is expected to undergo a quality review process before clinical or research use.^34^ Our results indicate that while fully manual segmentation can take from several hours up to a few days, quality review of model-generated segmentations can be completed within a few minutes or hours (Supplemental Materials 2.1), significantly reducing the overall annotation burden while maintaining reliability.

Several limitations to this study should be acknowledged. First, we had to rely on the accuracy of the trained readers because of the absence of a gold standard; thus, we used the inter-reader agreement as the target performance for the models. In addition, it was challenging even for the expert reader to delineate the cyst edges when the cysts presented as dense clusters with indistinct edges. It was observed that in the more severe testing cases, the DL-models had difficulty in detecting very small cysts (smaller than 1 ml). DL models require large amounts of data to achieve optimal results; thus, future work with a larger cohort is needed to further improve the performance of the models. This study used CT scans acquired only at our institution; therefore, larger multicenter studies would improve the generalizability of the DL-models. It is also noteworthy to acknowledge that the images used to train the model were limited to the contrast phase for the kidney cysts and noncontrast for the liver cysts. We used noncontrast CT scans for the instance cyst liver model as sufficient contrast exists between cysts and liver parenchyma to visualize the cyst edges. On the other hand, renal cysts and kidney parenchyma are more difficult to differentiate; thus, we used contrast-enhanced CT scans (nephrographic or corticomedullary phase) to train the kidney cyst segmentation model. Recent studies have made progress on using generative models to convert noncontrast CT scans of the kidneys into contrast-enhanced CTs. For instance, Pinnock *et al.*^35^ proposed a framework for generating multiphase synthetic contrast-enhanced CT scans of the kidney from noncontrast CT data. This may open the door for using our kidney cyst segmentation model on noncontrast CT scans and reduce the need for using contrast agents.

In summary, advanced imaging biomarkers can be derived from our automated approach and may have the potential to enhance the detection of early signs of polycystic kidney disease and PLD progression, paving the way for more personalized treatment approaches. However, the future advancement of disease characterization and management will require additional research to address the previous limitations and to validate these advanced methods and biomarkers through a large dataset and ensure their successful implementation in the clinical setting.

## Supplementary Material

## Data Availability

Original data generated for the study will be made available upon reasonable request to the corresponding author. Image Data. Public sharing of these data would be a violation of the Health Insurance Portability and Accountability Act (HIPAA) and the policies provided by our IRB related to this study.

## Declarative Statements

This study includes clinical experimentation and received Institutional Review Board or Ethics Committee approval. The need to obtain informed patient consent was waived. This study includes clinical experimentation and complies with the Declaration of Helsinki.

## Supplemental Material

Supplemental Methods

Supplemental Figure 1. Image preprocessing workflow. Conversion from instance-cyst segmentation level to semantic cyst-edge-core segmentation.

Supplemental Figure 2. (A) Example case showing 3 clusters of cysts indicated by labels 26, 12, and 1 with sphericity values of 0.48, 0.62, and 0.66, respectively. (B) Results of the watershed algorithm after each footprint iteration—the iterations stop after finding the sphericity peak or after reaching a plateau as in the case of label 1. (C) Result of the instance segmentation mask after watershed processing.

Supplemental Results

Supplemental Figure 3. (A–D) Absolute Bland–Altman analysis for total kidney cyst number between two independent readers (A), each reader with the DL model (B and C), and between the average of the readers with the DL model (D). (E–H) Linear regression analysis for total kidney cyst number between two independent readers (E), each reader with the DL model (F and G), and between the average of the readers with the DL model (H).

Supplemental Figure 4. (A–D) Absolute Bland–Altman analysis for total kidney cyst volume between two independent readers (A), each reader with the DL model (B and C), and between the average of the readers with the DL model (D). (E–H) Linear regression analysis for total kidney cyst volume between two independent readers (E), each reader with the DL model (F and G), and between the average of the readers with the DL model (H).

Supplemental Figure 5. (A–D) Absolute Bland–Altman analysis for CPSA between two independent readers (A), each reader with the DL model (B and C), and between the average of the readers with the DL model (D). (E–H) Linear regression analysis for CPSA between two independent readers (E), each reader with the DL model (F and G), and between the average of the readers with the DL model (H).

Supplemental Figure 6. (A–D) Absolute Bland–Altman analysis for total liver cyst number between two independent readers (A), each reader with the DL model (B and C), and between the average of the readers with the DL model (D). (E–H) Linear regression analysis for total liver cyst number between two independent readers (E), each reader with the DL model (F and G), and between the average of the readers with the DL model (H).

Supplemental Figure 7. (A–D) Absolute Bland–Altman analysis for total liver cyst volume between two independent readers (A), each reader with the DL model (B and C), and between the average of the readers with the DL model (D). (E–H) Linear regression analysis for total liver cyst volume between two independent readers (E), each reader with the DL model (F and G), and between the average of the readers with the DL model (H).

Supplemental Figure 8. Comparison of kidney cyst segmentation performance (dice score) across different stages of CKD, MIC, and TKV in patients with ADPKD. The red dots represent the interobserver agreement, and the blue and green dots represent the DL model with reader 1 and reader 2 agreements, respectively. The distribution of dice scores within each CKD stage, MIC, and TKV shows good to excellent agreement. A wider dice score distribution is observed in lower CKD, MIC, and TKV.

Supplemental Figure 9. Comparison of MIC and stages of CKD. Top row, the dot color represents the TCN value calculated from the segmentation performed by the expert reader, reader 2, and the DL model. Bottom row, the dot color represents the CPSA value in cm^2^ calculated from the segmentation performed by the expert reader, reader 2, and the DL model. It is observed that lower TCN and CPSA values associate with lower MIC and CKD stages, and higher TCN and CPSA values associate with higher MIC and CKD stages.
