# Supplementary material for: Deep Learning-Based Instance-Level Segmentation of Kidney and Liver Cysts in Computed Tomography Images of Patients Affected by Polycystic Kidney Disease
Source: Kidney360. 2025 Aug 14;7(1):117–30. doi: 10.34067/KID.0000000924 (PMC12889985; doi:10.34067/KID.0000000924)
Supplement: Supplementary file 1 [file kidney360-7-117-s001.pdf]

ASN American Society of Nephrology
JASN Journal of the American Society of Nephrology
KIDNEY360 Accessing Our World From Every Angle
CJASN Clinical Journal of the American Society of Nephrology

ASN Journal Disclosure Form

As per ASN journal policy, I have disclosed any financial relationships or commitments I have held in the past 36 months as included below. I have listed my Current Employer below to indicate there is a relationship requiring disclosure. If no relationship exists, my Current Employer is not listed.

F. Chebib reports the following:
Employer: Mayo Clinic; Research Funding: Research grant- Otsuka pharmaceuticals; Natera; Regulus; Vertex; and Patents or Royalties: Patent no US20200368191A1.

I understand that the information above will be published within the journal article, if accepted, and that failure to comply and/or to accurately and completely report the potential financial conflicts of interest could lead to the following: 1) Prior to publication, article rejection, or 2) Post-publication, sanctions ranging from, but not limited to, issuing a correction, reporting the inaccurate information to the authors' institution, banning authors from submitting work to ASN journals for varying lengths of time, and/or retraction of the published work.

Name: Fouad T. Chebib
Manuscript ID: K360-2025-000389R1
Manuscript Title: Deep-learning-based instance-level segmentation of kidney and liver cysts in CT images of patients affected by polycystic kidney Disease
Date of Completion: July 3, 2025
Disclosure Updated Date: May 8, 2025

ASN American Society of Nephrology
JASN Journal of the American Society of Nephrology
KIDNEY360 Accessing Our World From Every Angle
CJASN Clinical Journal of the American Society of Nephrology

ASN Journal Disclosure Form

As per ASN journal policy, I have disclosed any financial relationships or commitments I have held in the past 36 months as included below. I have listed my Current Employer below to indicate there is a relationship requiring disclosure. If no relationship exists, my Current Employer is not listed.

C. Cruz reports the following:
Employer: Fujifilm Cellular Dynamics

I understand that the information above will be published within the journal article, if accepted, and that failure to comply and/or to accurately and completely report the potential financial conflicts of interest could lead to the following: 1) Prior to publication, article rejection, or 2) Post-publication, sanctions ranging from, but not limited to, issuing a correction, reporting the inaccurate information to the authors' institution, banning authors from submitting work to ASN journals for varying lengths of time, and/or retraction of the published work.

Name: Conrad Cruz
Manuscript ID: K360-2025-000389R1
Manuscript Title: Deep-learning-based instance-level segmentation of kidney and liver cysts in CT images of patients affected by polycystic kidney Disease
Date of Completion: July 28, 2025
Disclosure Updated Date: May 14, 2025

ASN American Society of Nephrology | JASN Journal of the American Society of Nephrology | KIDNEY360 Accessing Our World From Every Angle | CJASN Clinical Journal of the American Society of Nephrology

ASN Journal Disclosure Form

As per ASN journal policy, I have disclosed any financial relationships or commitments I have held in the past 36 months as included below. I have listed my Current Employer below to indicate there is a relationship requiring disclosure. If no relationship exists, my Current Employer is not listed.

N. Dahl reports the following:
Employer: Mayo Clinic; Consultancy: Renasant Bio, Vertex, Regulus, Estuary Bio; Research Funding: I am a PI for clinical trials sponsored by Vertex and Regulus.; Advisory or Leadership Role: Natera Scientific Advisory Board; and Other Interests or Relationships: Associate Editor, Kidney360, Scientific Advisory Board, PKD Foundation.

I understand that the information above will be published within the journal article, if accepted, and that failure to comply and/or to accurately and completely report the potential financial conflicts of interest could lead to the following: 1) Prior to publication, article rejection, or 2) Post-publication, sanctions ranging from, but not limited to, issuing a correction, reporting the inaccurate information to the authors' institution, banning authors from submitting work to ASN journals for varying lengths of time, and/or retraction of the published work.

Name: Neera K. Dahl
Manuscript ID: K360-2025-000389R1
Manuscript Title: Deep-learning-based instance-level segmentation of kidney and liver cysts in CT images of patients affected by polycystic kidney Disease
Date of Completion: July 8, 2025
Disclosure Updated Date: July 8, 2025

ASN American Society of Nephrology | JASN Journal of the American Society of Nephrology | KIDNEY360 Accessing Our World From Every Angle | CJASN Clinical Journal of the American Society of Nephrology

ASN Journal Disclosure Form

As per ASN journal policy, I have disclosed any financial relationships or commitments I have held in the past 36 months as included below. I have listed my Current Employer below to indicate there is a relationship requiring disclosure. If no relationship exists, my Current Employer is not listed.

A. Denic reports the following:
Employer: Mayo Clinic

I understand that the information above will be published within the journal article, if accepted, and that failure to comply and/or to accurately and completely report the potential financial conflicts of interest could lead to the following: 1) Prior to publication, article rejection, or 2) Post-publication, sanctions ranging from, but not limited to, issuing a correction, reporting the inaccurate information to the authors' institution, banning authors from submitting work to ASN journals for varying lengths of time, and/or retraction of the published work.

Name: Aleksandar Denic
Manuscript ID: K360-2025-000389R1
Manuscript Title: Deep-learning-based instance-level segmentation of kidney and liver cysts in CT images of patients affected by polycystic kidney Disease
Date of Completion: July 3, 2025
Disclosure Updated Date: July 3, 2025

ASN American Society of Nephrology JASN Journal of the American Society of Nephrology KIDNEY360 Accessing Our World From Every Angle CJASN Clinical Journal of the American Society of Nephrology

ASN Journal Disclosure Form

As per ASN journal policy, I have disclosed any financial relationships or commitments I have held in the past 36 months as included below. I have listed my Current Employer below to indicate there is a relationship requiring disclosure. If no relationship exists, my Current Employer is not listed.

D. Elbarougy has nothing to disclose.

I understand that the information above will be published within the journal article, if accepted, and that failure to comply and/or to accurately and completely report the potential financial conflicts of interest could lead to the following: 1) Prior to publication, article rejection, or 2) Post-publication, sanctions ranging from, but not limited to, issuing a correction, reporting the inaccurate information to the authors' institution, banning authors from submitting work to ASN journals for varying lengths of time, and/or retraction of the published work.

Name: Doaa E. Elbarougy
Manuscript ID: K360-2025-000389R1
Manuscript Title: Deep-learning-based instance-level segmentation of kidney and liver cysts in CT images of patients affected by polycystic kidney Disease
Date of Completion: July 17, 2025
Disclosure Updated Date: July 15, 2025

ASN American Society of Nephrology | JASN Journal of the American Society of Nephrology | KIDNEY360 Accessing Our World From Every Angle | CJASN Clinical Journal of the American Society of Nephrology

ASN Journal Disclosure Form

As per ASN journal policy, I have disclosed any financial relationships or commitments I have held in the past 36 months as included below. I have listed my Current Employer below to indicate there is a relationship requiring disclosure. If no relationship exists, my Current Employer is not listed.

B. Erickson reports the following:
Employer: Mayo Clinic; Consultancy: HOPPR, AlphaSights; Ownership Interest: Enquanto.io, FlowSIGMA, Yunu, HOPPR; and Advisory or Leadership Role: Society for Imaging Informatics in Medicine; Radiology Society of North America.

I understand that the information above will be published within the journal article, if accepted, and that failure to comply and/or to accurately and completely report the potential financial conflicts of interest could lead to the following: 1) Prior to publication, article rejection, or 2) Post-publication, sanctions ranging from, but not limited to, issuing a correction, reporting the inaccurate information to the authors' institution, banning authors from submitting work to ASN journals for varying lengths of time, and/or retraction of the published work.

Name: Bradley J. Erickson
Manuscript ID: K360-2025-000389R1
Manuscript Title: "Deep-learning-based instance-level segmentation of kidney and liver cysts in CT images of patients affected by polycystic kidney Disease"
Date of Completion: July 8, 2025
Disclosure Updated Date: July 8, 2025

ASN
American Society of Nephrology
JASN
Journal of the American Society of Nephrology
KIDNEY360
Accessing Our World From Every Angle
CJASN
Clinical Journal of the American Society of Nephrology

ASN Journal Disclosure Form

As per ASN journal policy, I have disclosed any financial relationships or commitments I have held in the past 36 months as included below. I have listed my Current Employer below to indicate there is a relationship requiring disclosure. If no relationship exists, my Current Employer is not listed.

A. Gregory reports the following:
Employer: Mayo Clinic; and Patents or Royalties: Mayo Clinic.

I understand that the information above will be published within the journal article, if accepted, and that failure to comply and/or to accurately and completely report the potential financial conflicts of interest could lead to the following: 1) Prior to publication, article rejection, or 2) Post-publication, sanctions ranging from, but not limited to, issuing a correction, reporting the inaccurate information to the authors' institution, banning authors from submitting work to ASN journals for varying lengths of time, and/or retraction of the published work.

Name: Adriana Gregory
Manuscript ID: K360-2025-000389R1
Manuscript Title: Deep-learning-based instance-level segmentation of kidney and liver cysts in CT images of patients affected by polycystic kidney Disease
Date of Completion: July 17, 2025
Disclosure Updated Date: May 8, 2025

ASN American Society of Nephrology | JASN Journal of the American Society of Nephrology | KIDNEY360 Accessing Our World From Every Angle | CJASN Clinical Journal of the American Society of Nephrology

ASN Journal Disclosure Form

As per ASN journal policy, I have disclosed any financial relationships or commitments I have held in the past 36 months as included below. I have listed my Current Employer below to indicate there is a relationship requiring disclosure. If no relationship exists, my Current Employer is not listed.

P. Harris reports the following:
Employer: Mayo Clinic; Consultancy: Vertex, Mitobridge, Regulus, Janssen, Maze Therapeutics, Caraway Therapeutics, Renasant, Sen Therapeutics; PYC Therapeutics, Arnatar Therapeutics, Torque Bio, RA Ventures; Research Funding: Espervita, Janssen, Acceleron (Merck), Jemincare, Regulus (Novartis), PYC; and Patents or Royalties: Bayer, Maze Therapeutics, Calico Life Sciences, Sonothera, Inc. Torque Bio, Arnatar Therapeutics, Vertex Pharmaceuticals.

I understand that the information above will be published within the journal article, if accepted, and that failure to comply and/or to accurately and completely report the potential financial conflicts of interest could lead to the following: 1) Prior to publication, article rejection, or 2) Post-publication, sanctions ranging from, but not limited to, issuing a correction, reporting the inaccurate information to the authors' institution, banning authors from submitting work to ASN journals for varying lengths of time, and/or retraction of the published work.

Name: Peter C. Harris
Manuscript ID: K360-2025-000389R1
Manuscript Title: Deep-learning-based instance-level segmentation of kidney and liver cysts in CT images of patients affected by polycystic kidney Disease
Date of Completion: July 31, 2025
Disclosure Updated Date: July 21, 2025

ASN American Society of Nephrology | JASN Journal of the American Society of Nephrology | KIDNEY360 Accessing Our World From Every Angle | CJASN Clinical Journal of the American Society of Nephrology

ASN Journal Disclosure Form

As per ASN journal policy, I have disclosed any financial relationships or commitments I have held in the past 36 months as included below. I have listed my Current Employer below to indicate there is a relationship requiring disclosure. If no relationship exists, my Current Employer is not listed.

M. Hogan reports the following:
Employer: Mayo Clinic; Research Funding: Camurus, ; Regulus Pharmaceuticals ; Reata; IC Medtech; Advisory or Leadership Role: Mayo Clinic Proceedings Quality & Outcomes Journal; No payment; Camurus Pharmaceuticals.; No payment; Sail Bio, No payment.; Glaxo-Smith-Kline, No payment. American Society of Nephrology, no payment. Regulus - no payment. Glaxo Smith Kline no payment.; and Other Interests or Relationships: PKD Foundation; PKD Disease Outcomes Consortium; American Society of Nephrology;.

I understand that the information above will be published within the journal article, if accepted, and that failure to comply and/or to accurately and completely report the potential financial conflicts of interest could lead to the following: 1) Prior to publication, article rejection, or 2) Post-publication, sanctions ranging from, but not limited to, issuing a correction, reporting the inaccurate information to the authors' institution, banning authors from submitting work to ASN journals for varying lengths of time, and/or retraction of the published work.

Name: Marie C. Hogan
Manuscript ID: K360-2025-000389R1
Manuscript Title: Deep-learning-based instance-level segmentation of kidney and liver cysts in CT images of patients affected by polycystic kidney Disease
Date of Completion: July 3, 2025
Disclosure Updated Date: July 3, 2025

ASN
American Society of Nephrology
JASN
Journal of the American Society of Nephrology
KIDNEY360
Accessing Our World From Every Angle
CJASN
Clinical Journal of the American Society of Nephrology

ASN Journal Disclosure Form

As per ASN journal policy, I have disclosed any financial relationships or commitments I have held in the past 36 months as included below. I have listed my Current Employer below to indicate there is a relationship requiring disclosure. If no relationship exists, my Current Employer is not listed.

J. Im reports the following:
Employer: Hackensack Meridian School of Medicine

I understand that the information above will be published within the journal article, if accepted, and that failure to comply and/or to accurately and completely report the potential financial conflicts of interest could lead to the following: 1) Prior to publication, article rejection, or 2) Post-publication, sanctions ranging from, but not limited to, issuing a correction, reporting the inaccurate information to the authors' institution, banning authors from submitting work to ASN journals for varying lengths of time, and/or retraction of the published work.

Name: Jeeho Im
Manuscript ID: K360-2025-000389R1
Manuscript Title: Deep-learning-based instance-level segmentation of kidney and liver cysts in CT images of patients affected by polycystic kidney Disease
Date of Completion: July 17, 2025
Disclosure Updated Date: July 17, 2025

ASN American Society of Nephrology
JASN Journal of the American Society of Nephrology
KIDNEY360 Accessing Our World From Every Angle
CJASN Clinical Journal of the American Society of Nephrology

ASN Journal Disclosure Form

As per ASN journal policy, I have disclosed any financial relationships or commitments I have held in the past 36 months as included below. I have listed my Current Employer below to indicate there is a relationship requiring disclosure. If no relationship exists, my Current Employer is not listed.

M. Khalifa has nothing to disclose.

I understand that the information above will be published within the journal article, if accepted, and that failure to comply and/or to accurately and completely report the potential financial conflicts of interest could lead to the following: 1) Prior to publication, article rejection, or 2) Post-publication, sanctions ranging from, but not limited to, issuing a correction, reporting the inaccurate information to the authors' institution, banning authors from submitting work to ASN journals for varying lengths of time, and/or retraction of the published work.

Name: Muhammed Khalifa
Manuscript ID: K360-2025-000389R1
Manuscript Title: Deep-learning-based instance-level segmentation of kidney and liver cysts in CT images of patients affected by polycystic kidney Disease
Date of Completion: July 17, 2025
Disclosure Updated Date: July 17, 2025

ASN American Society of Nephrology JASN Journal of the American Society of Nephrology KIDNEY360 Accessing Our World From Every Angle CJASN Clinical Journal of the American Society of Nephrology

ASN Journal Disclosure Form

As per ASN journal policy, I have disclosed any financial relationships or commitments I have held in the past 36 months as included below. I have listed my Current Employer below to indicate there is a relationship requiring disclosure. If no relationship exists, my Current Employer is not listed.

T. Kline reports the following:
Employer: Mayo Clinic; Consultancy: Regulus Therapeutics, Inc.; Vertex Pharmaceuticals, Inc.; GlaxoSmithKline ; PYC Therapeutics; Research Funding: Regulus Therapeutics, Inc.; and Patents or Royalties: Disclosure at Mayo Clinic that is currently licensed by Regulus.

I understand that the information above will be published within the journal article, if accepted, and that failure to comply and/or to accurately and completely report the potential financial conflicts of interest could lead to the following: 1) Prior to publication, article rejection, or 2) Post-publication, sanctions ranging from, but not limited to, issuing a correction, reporting the inaccurate information to the authors' institution, banning authors from submitting work to ASN journals for varying lengths of time, and/or retraction of the published work.

Name: Timothy L. Kline
Manuscript ID: K360-2025-000389R1
Manuscript Title: Deep-learning-based instance-level segmentation of kidney and liver cysts in CT images of patients affected by polycystic kidney Disease
Date of Completion: July 9, 2025
Disclosure Updated Date: July 24, 2024

ASN American Society of Nephrology JASN Journal of the American Society of Nephrology KIDNEY360 Accessing Our World From Every Angle CJASN Clinical Journal of the American Society of Nephrology

ASN Journal Disclosure Form

As per ASN journal policy, I have disclosed any financial relationships or commitments I have held in the past 36 months as included below. I have listed my Current Employer below to indicate there is a relationship requiring disclosure. If no relationship exists, my Current Employer is not listed.

T. Potretzke has nothing to disclose.

I understand that the information above will be published within the journal article, if accepted, and that failure to comply and/or to accurately and completely report the potential financial conflicts of interest could lead to the following: 1) Prior to publication, article rejection, or 2) Post-publication, sanctions ranging from, but not limited to, issuing a correction, reporting the inaccurate information to the authors' institution, banning authors from submitting work to ASN journals for varying lengths of time, and/or retraction of the published work.

Name: Theodora A. Potretzke
Manuscript ID: K360-2025-000389R1
Manuscript Title: Deep-learning-based instance-level segmentation of kidney and liver cysts in CT images of patients affected by polycystic kidney Disease
Date of Completion: July 22, 2025
Disclosure Updated Date: May 14, 2025

ASN American Society of Nephrology JASN Journal of the American Society of Nephrology KIDNEY360 Accessing Our World From Every Angle CJASN Clinical Journal of the American Society of Nephrology

ASN Journal Disclosure Form

As per ASN journal policy, I have disclosed any financial relationships or commitments I have held in the past 36 months as included below. I have listed my Current Employer below to indicate there is a relationship requiring disclosure. If no relationship exists, my Current Employer is not listed.

S. Ramanathan reports the following:
Employer: Mayo clinic

I understand that the information above will be published within the journal article, if accepted, and that failure to comply and/or to accurately and completely report the potential financial conflicts of interest could lead to the following: 1) Prior to publication, article rejection, or 2) Post-publication, sanctions ranging from, but not limited to, issuing a correction, reporting the inaccurate information to the authors' institution, banning authors from submitting work to ASN journals for varying lengths of time, and/or retraction of the published work.

Name: Sumana Ramanathan
Manuscript ID: K360-2025-000389R1
Manuscript Title: Deep-learning-based instance-level segmentation of kidney and liver cysts in CT images of patients affected by polycystic kidney Disease,
Date of Completion: July 17, 2025
Disclosure Updated Date: July 17, 2025

ASN American Society of Nephrology JASN Journal of the American Society of Nephrology KIDNEY360 Accessing Our World From Every Angle CJASN Clinical Journal of the American Society of Nephrology

ASN Journal Disclosure Form

As per ASN journal policy, I have disclosed any financial relationships or commitments I have held in the past 36 months as included below. I have listed my Current Employer below to indicate there is a relationship requiring disclosure. If no relationship exists, my Current Employer is not listed.

A. Rule reports the following:
Employer: Mayo Clinic; Patents or Royalties: UpToDate; and Advisory or Leadership Role: JASN - Associate Editon; Mayo Clinic Proceedings - Section Editor.

I understand that the information above will be published within the journal article, if accepted, and that failure to comply and/or to accurately and completely report the potential financial conflicts of interest could lead to the following: 1) Prior to publication, article rejection, or 2) Post-publication, sanctions ranging from, but not limited to, issuing a correction, reporting the inaccurate information to the authors' institution, banning authors from submitting work to ASN journals for varying lengths of time, and/or retraction of the published work.

Name: Andrew D. Rule
Manuscript ID: K360-2025-000389R1
Manuscript Title: Deep-learning-based instance-level segmentation of kidney and liver cysts in CT images of patients affected by polycystic kidney Disease
Date of Completion: July 3, 2025
Disclosure Updated Date: October 4, 2024

ASN American Society of Nephrology
JASN Journal of the American Society of Nephrology
KIDNEY360 Accessing Our World From Every Angle
CJASN Clinical Journal of the American Society of Nephrology

ASN Journal Disclosure Form

As per ASN journal policy, I have disclosed any financial relationships or commitments I have held in the past 36 months as included below. I have listed my Current Employer below to indicate there is a relationship requiring disclosure. If no relationship exists, my Current Employer is not listed.

V. Torres reports the following:
Employer: Mayo Clinic; Research Funding: Mironid, Tribune Therapeutics, GSK, Regulus [all payments to Mayo Foundation for Preclinical and clinical trials and preclinical research]; Honoraria: Up to Date; Patents or Royalties: Consulting agreement with uResearch Technology and MFMER for imaging analytics for PCKD.; Repurposing of probenecid to treat PCKD.; System and method of classifying ADPKD; and Advisory or Leadership Role: International Society of Nephrology (Kaplan award committee), American Society of Nephrology (editorial board), PKD Foundation advisory board.

I understand that the information above will be published within the journal article, if accepted, and that failure to comply and/or to accurately and completely report the potential financial conflicts of interest could lead to the following: 1) Prior to publication, article rejection, or 2) Post-publication, sanctions ranging from, but not limited to, issuing a correction, reporting the inaccurate information to the authors' institution, banning authors from submitting work to ASN journals for varying lengths of time, and/or retraction of the published work.

Name: Vicente E. Torres
Manuscript ID: K360-2025-000389R1
Manuscript Title: Deep-learning-based instance-level segmentation of kidney and liver cysts in CT images of patients affected by polycystic kidney Disease
Date of Completion: July 31, 2025
Disclosure Updated Date: June 12, 2025

ASN
American Society of Nephrology
JASN
Journal of the American Society of Nephrology
KIDNEY360
Accessing Our World From Every Angle
CJASN
Clinical Journal of the American Society of Nephrology

ASN Journal Disclosure Form

As per ASN journal policy, I have disclosed any financial relationships or commitments I have held in the past 36 months as included below. I have listed my Current Employer below to indicate there is a relationship requiring disclosure. If no relationship exists, my Current Employer is not listed.

H. Yang reports the following:
Employer: Mayo Clinic

I understand that the information above will be published within the journal article, if accepted, and that failure to comply and/or to accurately and completely report the potential financial conflicts of interest could lead to the following: 1) Prior to publication, article rejection, or 2) Post-publication, sanctions ranging from, but not limited to, issuing a correction, reporting the inaccurate information to the authors' institution, banning authors from submitting work to ASN journals for varying lengths of time, and/or retraction of the published work.

Name: Hana Yang
Manuscript ID: K360-2025-000389R1
Manuscript Title: Deep-learning-based instance-level segmentation of kidney and liver cysts in CT images of patients affected by polycystic kidney Disease,
Date of Completion: July 3, 2025
Disclosure Updated Date: December 31, 2024
