# Supplementary material for: Deep Learning-Based Instance-Level Segmentation of Kidney and Liver Cysts in Computed Tomography Images of Patients Affected by Polycystic Kidney Disease
Source: Kidney360. 2025 Aug 14;7(1):117–30. doi: 10.34067/KID.0000000924 (PMC12889985; doi:10.34067/KID.0000000924)
Supplement: Supplementary file 2 [file kidney360-7-117-s002.pdf]

Supplemental Material

1. Supplemental Methods

1.1. Active Learning Training Strategy and Data Labeling

The quality assessment time was recorded for comparison between stages. Inference on additional training images was done using the preliminary DL-models. The predicted segmentations were checked for accuracy and used to retrain the DL segmentation models.

Fifty training examples (25 kidney and 25 liver cases) and the test sets were first labeled. Semantic kidney- and liver-cyst segmentation were done manually utilizing the software ITK-Snap. To convert the semantic segmentation into instance cyst segmentations, a 3D connected component algorithm with 6-nearest neighbors was used to assign the instance labels, followed by a modified 3D watershed algorithm to split connected cysts (post-processing method section 1.3).

1.2. Pre-processing: Semantic to Instance Conversion

The CT scans and corresponding cyst segmentations were up-sampled to 512x512x3*SliceNumber matrix size by bicubic and nearest neighbor interpolations, respectively. The number of slices were increased using a factor of 3 to prevent the removal of cysts appearing in a single slice during preprocessing. The instance segmentations were converted to cyst-edge and cyst-core masks by applying the following preprocessing in a cyst-by-cyst fashion. First, a 3D erosion algorithm with a 6-connected structuring element was computed to generate the cyst-core mask. Then, the eroded mask was subtracted from the binarized original cyst segmentation to generate the cyst-edge mask. The edge and core masks were combined assigning a label of 1 to the core voxels and a label of 2 to the edge voxels. Finally, the edge-core mask was combined with the kidney or liver mask computed with a label of 3. Supplemental figure 1 depicts this process.

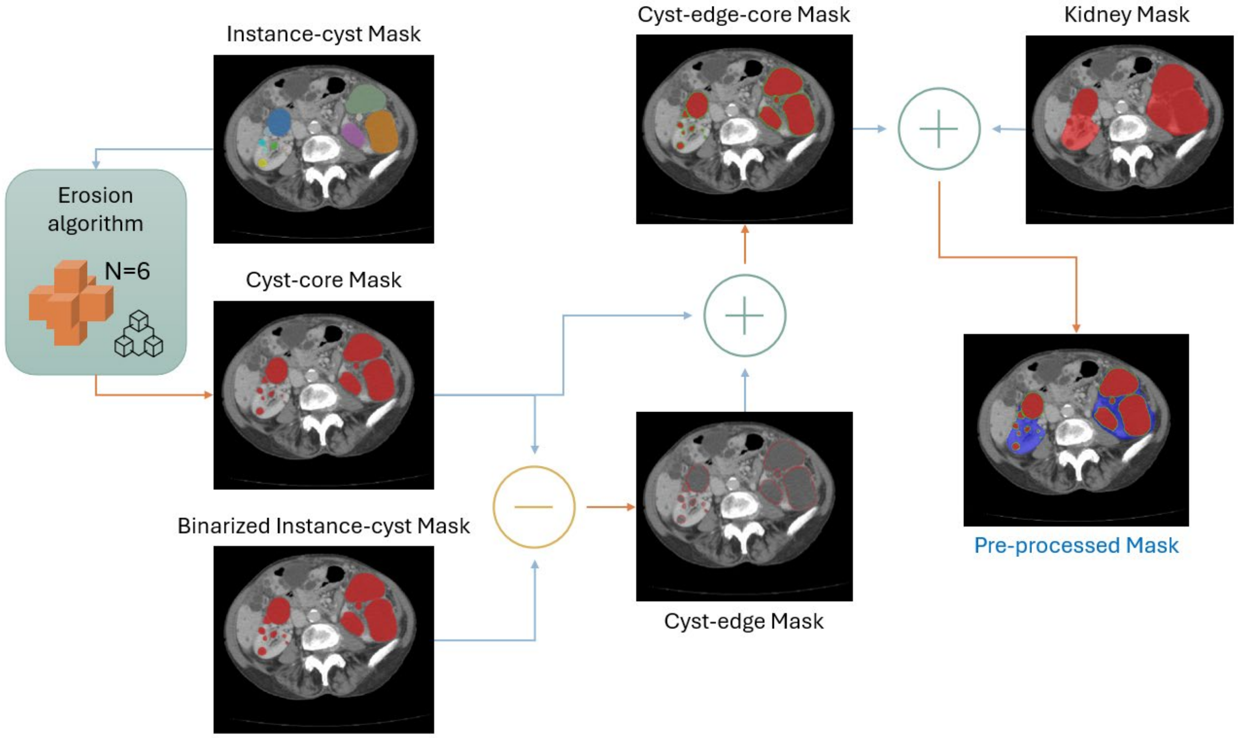

Supplemental Figure 1. Image pre-processing workflow. Conversion from instance-cyst segmentation level to semantic cyst-edge-core segmentation.

1.3. Post-processing: Modified Watershed Method

To convert the cyst edge-core masks back into instance cyst segmentations, first the core label was split into multiple labels by utilizing a connected components algorithm. Then, each label was assessed in terms of a sphericity score defined as:

$$sphericity = \frac{\sqrt[3]{36\pi V^2}}{A}$$

Where V is the volume and A is the surface area of the cyst label. The sphericity values range from 0 to 1, where 1 indicates a perfect sphere. Recognizing that cysts are often spherical, we optimized the post-processing algorithm using the sphericity score. Labels with a sphericity score below 0.8 were considered as potential clustered cysts that night have not been split completely by the predicted cyst-edges. A modified watershed algorithm was applied to separate clusters of cysts utilizing the sphericity score as an optimization parameter for the algorithm's footprint. The footprint was initialized as the average of the maximum diameters in the x, y, and z directions. Supplemental figure 2 depicts an example case with some clustered cysts that were successfully separated by the modified watershed algorithm.

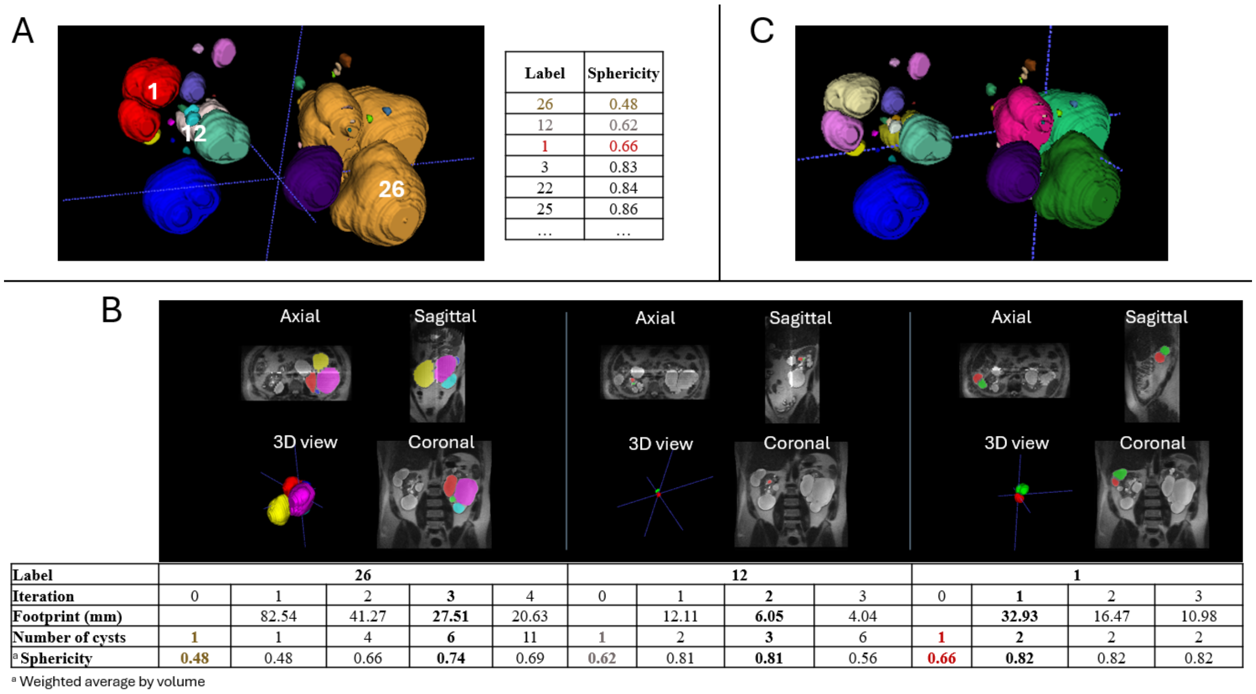

Label	Sphericity
26	0.48
12	0.62
1	0.66
3	0.83
22	0.84
25	0.86
…	…

Label	26					12				1			
Iteration	0	1	2	**3**	4	0	1	**2**	3	0	**1**	2	3
Footprint (mm)		82.54	41.27	**27.51**	20.63		12.11	**6.05**	4.04		**32.93**	16.47	10.98
Number of cysts	**1**	1	4	**6**	11	**1**	2	**3**	6	**1**	**2**	2	2
[a] Sphericity	**0.48**	0.48	0.66	**0.74**	0.69	**0.62**	0.81	**0.81**	0.56	**0.66**	**0.82**	0.82	0.82

[a] Weighted average by volume

Supplemental Figure 2. (A) Example case showing 3 clusters of cysts indicated by labels 26, 12, and 1 with sphericity values of 0.48, 0.62 and 0.66, respectively. (B) Results of the watershed algorithm after each footprint iteration, the iterations stop after finding the sphericity peak or after reaching a plateau as in the case of label 1. (C) Result of the instance segmentation mask after watershed processing.

To preserve all the cyst cores that might have been lost after this post-processing, a 6-nearest connectivity based connected components algorithm was applied to the difference between the predicted core distance map and the 3D watershed output. Finally, a 3x3x3 voxel dilation with 6-connected structuring element was performed on all the cyst instances, and the images were down-sampled back to their original corresponding CT shapes.

1.4. Nascimento Nomenclature Methods

This methodology was applied to compare cyst segmentations in both the kidney and liver across multiple segmentation masks, with pairwise comparisons between DL segmentations and those produced by two readers. Additionally, inter-rater comparisons were performed to assess the agreement between the two readers.

1.4.1. Data Preparation

Segmentation masks for each case were stored as NIfTI files and categorized into DL-generated and manually labeled datasets for each reader. A program was developed to calculate the Nascimento nomenclature metrics, which include Correct Detections (CD), False Positives (FP), False Negatives (FN), Splits, Merges, and Split-Merges (see graphical depiction in Figure 2). The script processes the segmentation data by loading the DL and manual masks, followed by identifying cyst labels based on unique non-background values in the segmentation masks.

1.4.2. Overlap Matrix Construction

An overlap matrix was constructed for each pair of DL and manual segmentation masks. The matrix quantifies the voxel-level overlap between each cyst in the DL segmentation and each cyst in the manual segmentation. The overlap is computed for all unique cyst labels, excluding the background. This matrix served as the basis for further metric calculations, including the computation of Dice coefficients for each cyst pair.

1.4.3. Metric Calculation

The Nascimento nomenclature metrics were calculated based on the overlap matrix. Specifically:

- **Correct Detections (CD)** were defined as cysts that had a one-to-one match between the DL and manual segmentations based on a Dice coefficient threshold of 0.1.
- **False Positives (FP)** were defined as cysts detected by the DL segmentation but not present in the manual segmentation.
- **False Negatives (FN)** were cysts present in the manual segmentation but not detected by the DL segmentation.
- **Splits** were counted when a single manual cyst was segmented as multiple cysts in the DL segmentation.
- **Merges** were counted when multiple manual cysts were merged into a single cyst by the DL segmentation.
- **Split-Merges** were identified when both splits and merges occurred between corresponding cysts in the DL and manual segmentations, and corrections were made to avoid double-counting these instances.

2. Supplemental Results

2.1. Active-Learning Quality Review

The time required for quality checking the images after the first DL-model training stage ranged from 2 hours to 30 hours for ADPKD/PLD cases, with an average time of 16 hours/case. However, after the second iteration of model retraining, the time required for quality checking the images was significantly reduced to around 10 minutes to 4 hours for ADPKD/PLD cases with an average time of 2 hours/case.

2.2. Nascimento Nomenclature Results Summary

The DL segmentations exhibited comparable performance against both readers, with minor variations between the two organs. Correct detections and false negatives tended to be higher for kidneys than for liver, while the liver had more frequent splits and split-merges. Interobserver comparisons showed higher correct detections and generally lower false positive and false negative counts compared to DL comparisons, indicating stronger agreement between the two human readers.

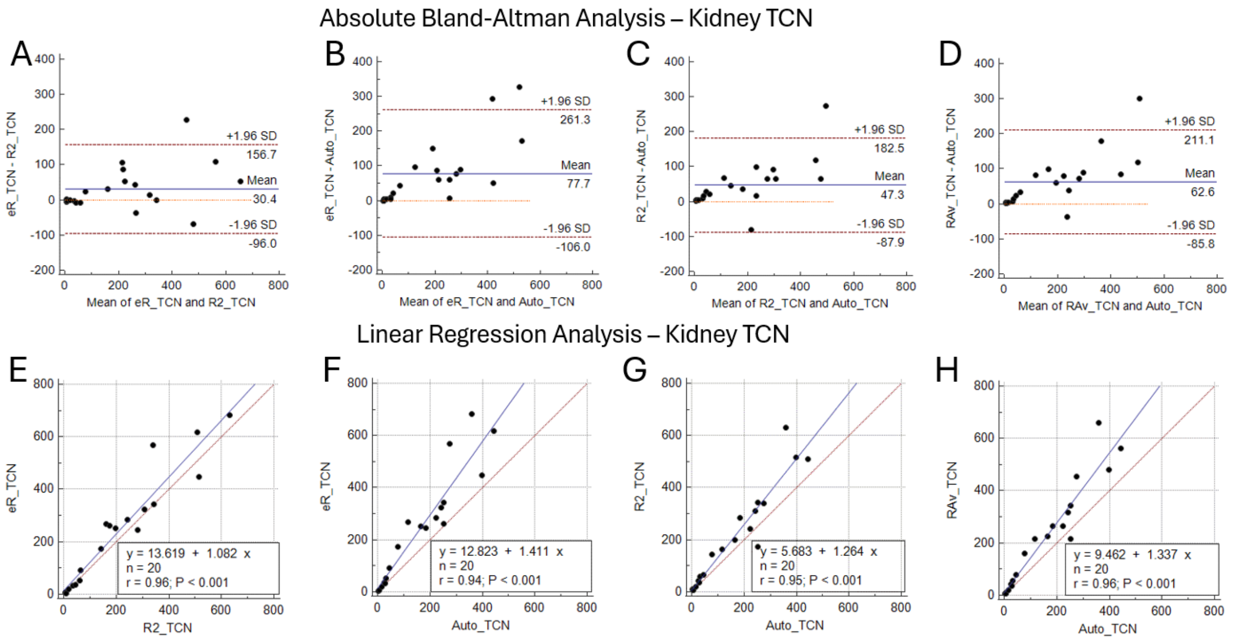

Supplemental Figure 3. (A-D) Absolute Bland-Altman analysis for total kidney cyst number between two independent readers (A), each reader with the DL model (B, C), and between the average of the readers with the DL model (D). (E-H) Linear regression analysis for total kidney cyst number between two independent readers (E), each reader with the DL model (F, G), and between the average of the readers with the DL model (H).

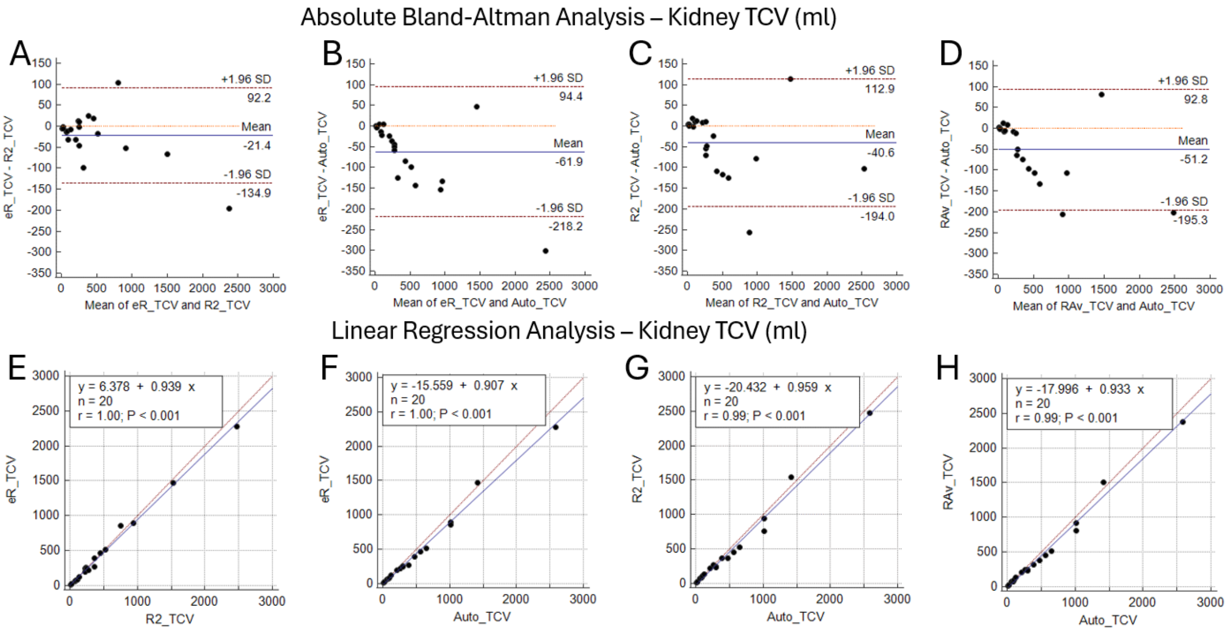

Supplemental Figure 4. (A-D) Absolute Bland-Altman analysis for total kidney cyst volume between two independent readers (A), each reader with the DL model (B, C), and between the average of the readers with the DL model (D). (E-H) Linear regression analysis for total kidney cyst volume between two independent readers (E), each reader with the DL model (F, G), and between the average of the readers with the DL model (H).

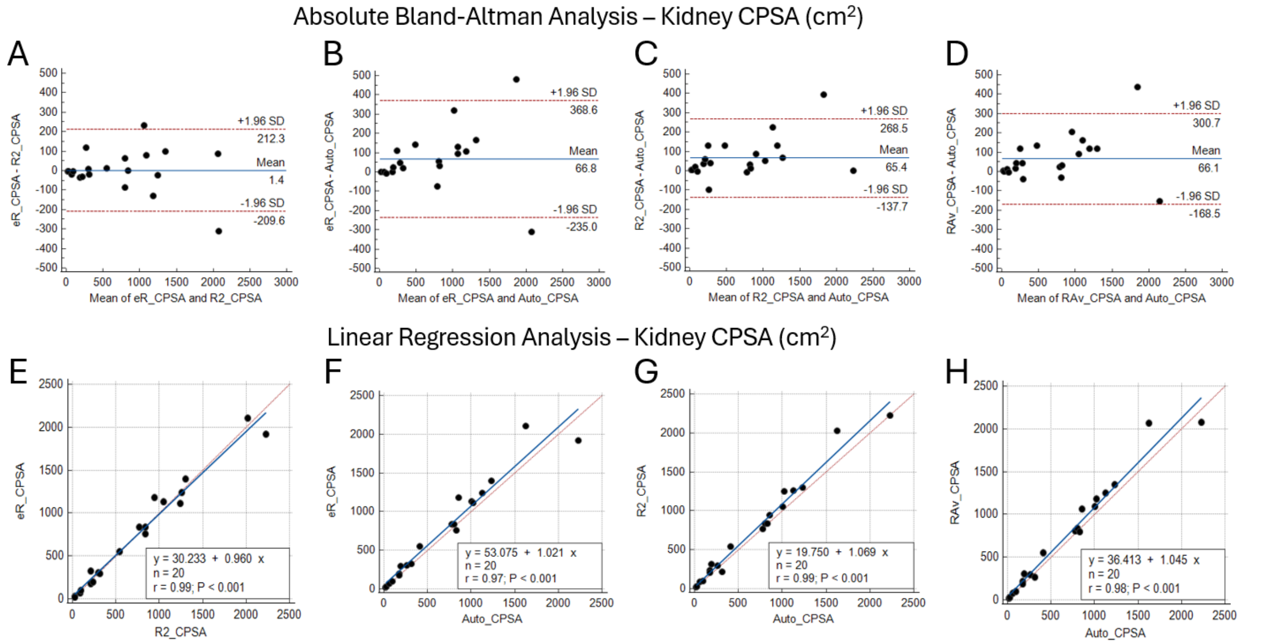

Supplemental Figure 5. (A-D) Absolute Bland-Altman analysis for cyst-parenchyma surface area between two independent readers (A), each reader with the DL model (B, C), and between the average of the readers with the DL model (D). (E-H) Linear regression analysis for cyst-parenchyma surface area between two independent readers (E), each reader with the DL model (F, G), and between the average of the readers with the DL model (H).

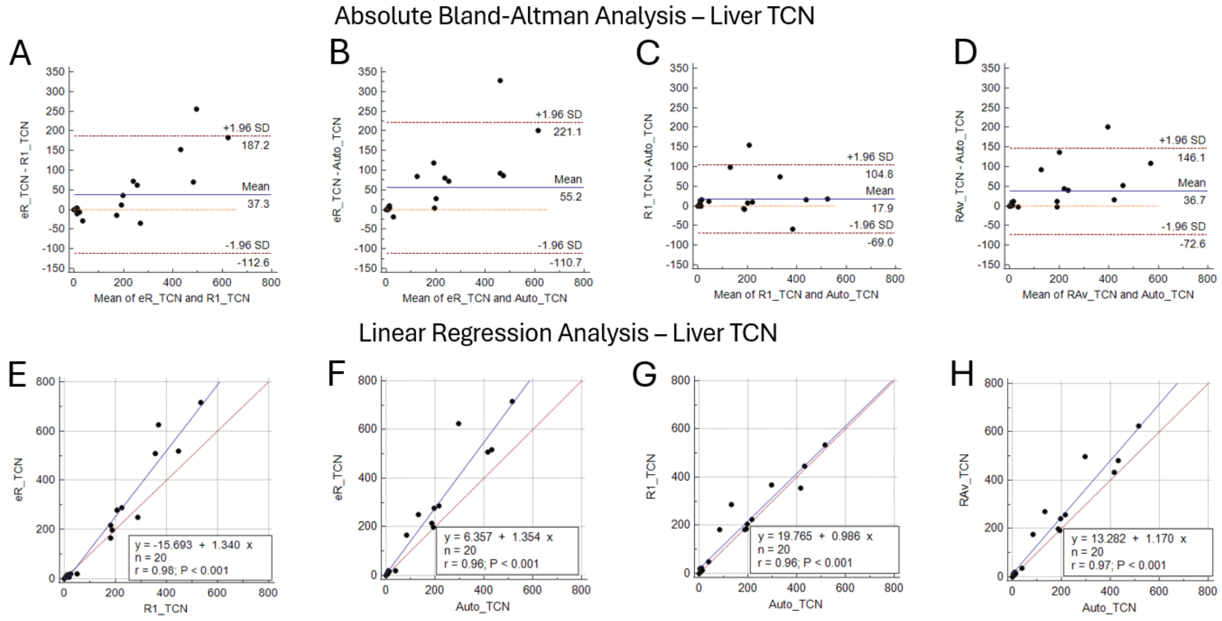

Supplemental Figure 6. (A-D) Absolute Bland-Altman analysis for total liver cyst number between two independent readers (A), each reader with the DL model (B, C), and between the average of the readers with the DL model (D). (E-H) Linear regression analysis for total liver cyst number between two independent readers (E), each reader with the DL model (F, G), and between the average of the readers with the DL model (H).

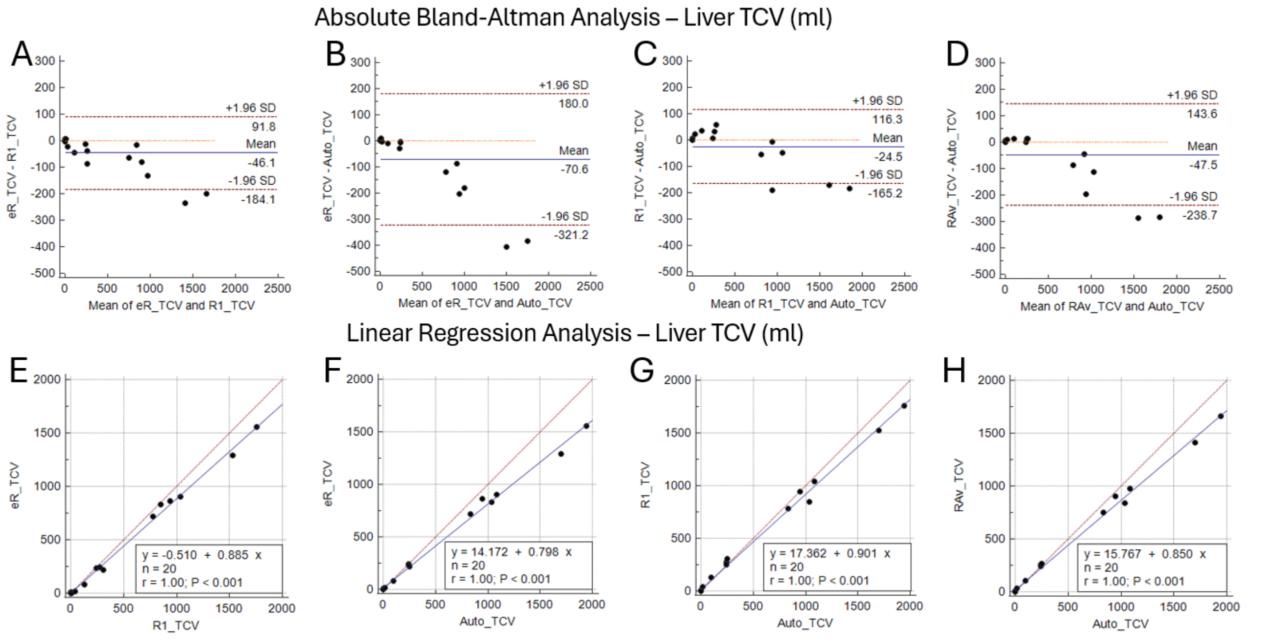

Supplemental Figure 7. (A-D) Absolute Bland-Altman analysis for total liver cyst volume between two independent readers (A), each reader with the DL model (B, C), and between the average of the readers with the DL model (D). (E-H) Linear regression analysis for total liver cyst volume between two independent readers (E), each reader with the DL model (F, G), and between the average of the readers with the DL model (H).

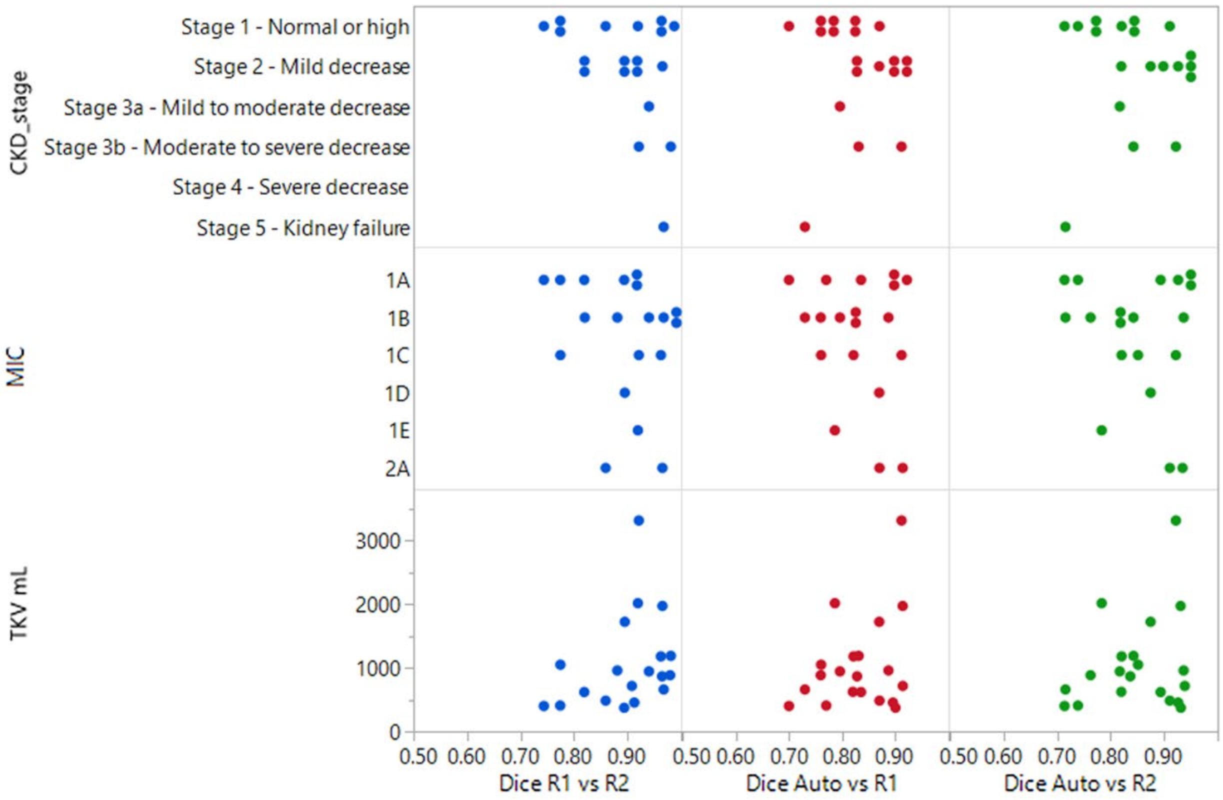

Supplemental Figure 8. Comparison of kidney cyst segmentation performance (Dice score) across different stages of chronic kidney disease (CKD), Mayo Imaging Classes (MIC), and total kidney volumes (TKV) in ADPKD patients. The red dots represent the inter-observer agreement, and the blue and green dots represent the DL model with reader 1 and reader 2 agreements, respectively. The distribution of Dice scores within each CKD stage, MIC, and TKV shows good to excellent agreement. A wider Dice score distribution is observed in lower CKD, MIC, and TKV.

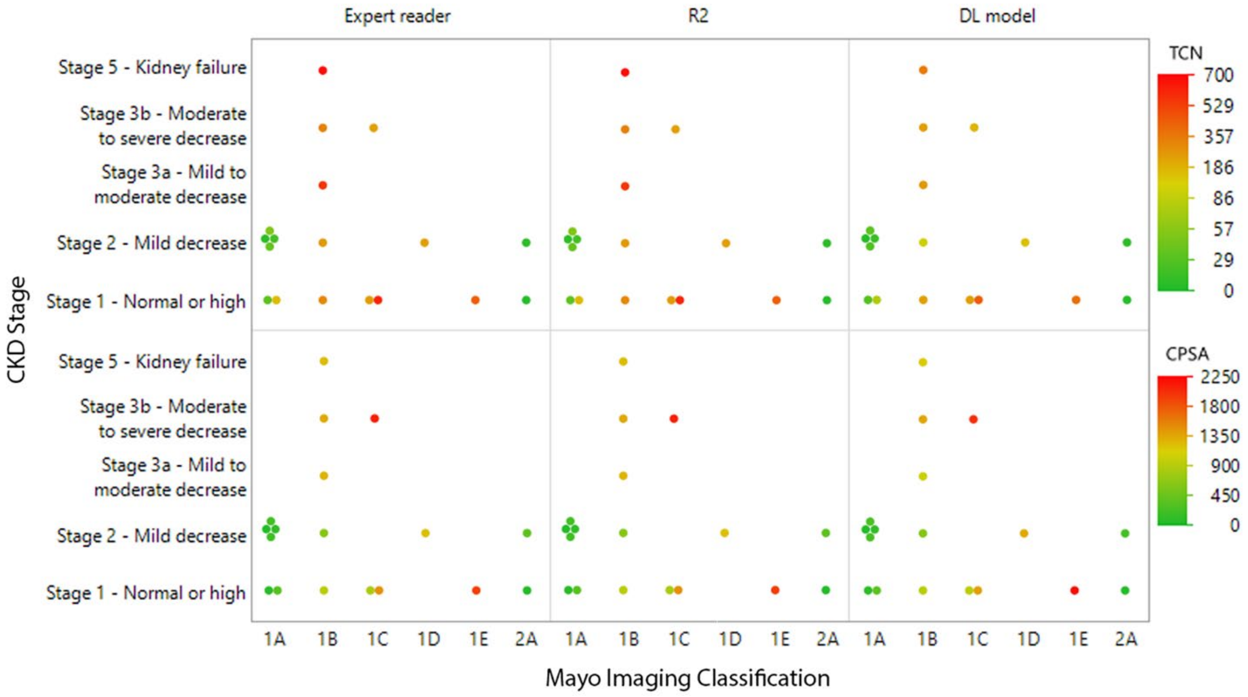

Supplemental Figure 9. Comparison of Mayo Imaging Classification (MIC) and stages of chronic kidney disease (CKD). Top row, the dot color represents the total cyst number (TCN) value calculated from the segmentation performed by the expert reader, reader 2, and the DL model. Bottom row, the dot color represents the cyst-parenchyma surface area (CPSA) value in cm^2 calculated from the segmentation performed by the expert reader, reader 2, and the DL model. It is observed that lower TCN and CPSA values associate with lower MIC and CKD stages, and higher TCN and CPSA values associate with higher MIC and CKD stages.
